# Risk prediction of bronchopulmonary dysplasia in preterm infants by the nomogram model

**DOI:** 10.3389/fped.2023.1117142

**Published:** 2023-03-14

**Authors:** Yang Gao, Dongyun Liu, Yingmeng Guo, Menghan Cao

**Affiliations:** ^1^ Department of Neonatology, Linyi Central Hospital, Linyi, China; ^2^ Department of Pediatrics, Affiliated Hospital of Qingdao University, Qingdao, China

**Keywords:** BPD, logistic regression analysis, risk prediction, preterm infants, nomogram model

## Abstract

**Backgrounds and Aims:**

Bronchopulmonary dysplasia (BPD) has serious immediate and long-term sequelae as well as morbidity and mortality. The objective of this study is to develop a predictive model of BPD for premature infants using clinical maternal and neonatal parameters.

**Methods:**

This single-center retrospective study enrolled 237 cases of premature infants with gestational age less than 32 weeks. The research collected demographic, clinical and laboratory parameters. Univariate logistic regression analysis was carried out to screen the potential risk factors of BPD. Multivariate and LASSO logistic regression analysis was performed to further select variables for the establishment of nomogram models. The discrimination of the model was assessed by C-index. The Hosmer-Lemeshow test was used to assess the calibration of the model.

**Results:**

Multivariate analysis identified maternal age, delivery option, neonatal weight and age, invasive ventilation, and hemoglobin as risk predictors. LASSO analysis selected delivery option, neonatal weight and age, invasive ventilation, hemoglobin and albumin as the risk predictors. Both multivariate (AUC = 0.9051; HL *P* = 0.6920; C-index = 0.910) and LASSO (AUC = 0.8935; HL *P* = 0.7796; C-index = 0.899) - based nomograms exhibited ideal discrimination and calibration as confirmed by validation dataset.

**Conclusions:**

The probability of BPD in a premature infant could be effectively predicted by the nomogram model based on the clinical maternal and neonatal parameters. However, the model required external validation using larger samples from multiple medical centers.

## Introduction

Bronchopulmonary dysplasia (BPD) is a common chronic lung disease in preterm infants, leading to long-term complications such as cardiopulmonary dysfunction and growth retardation (1, 2). In particular, moderate and severe BPD is a major cause of death and neurodevelopmental disability in preterm infants (3, 4). Although the survival rate of preterm infants has increased significantly with the widespread application of antenatal steroids, exogenous pulmonary surfactant replacement therapy, and the development of neonatal intensive care, the incidence of BPD has been not dramatically decreased (5–7). What's more, BPD has serious immediate and long-term sequelae as well as morbidity and mortality (8–10). Therefore, the prevention of BPD has become a hot topic of clinical concern.

The pathogenesis of BPD is complex, with the underlying cause being impaired development of the immature lung in response to inflammation, hyperoxia, and other damaging factors (11, 12). Prenatal and neonatal factors have been associated with BPD (13, 14). The prenatal factors are related to the lack of antenatal steroid therapy, chorioamnionitis, and maternal hypertension (13, 14). Neonatal factors include gestational age, birth weight, and postdelivery resuscitation (13, 14). The independent prenatal risk factors may be oligohydramnios, male gender, and intrauterine growth restriction (14, 15). Postnatal risk factors seem to be the length of exposure to mechanical ventilation, nosocomial pneumonia, and the necessity for FiO_2_ of more than 0.30 in the delivery room (14, 15). It is important to prevent and control the occurrence of BPD if the risk of BPD development can be identified and assessed early.

The statistical analysis methods commonly used to select the risk variables contain the logistic regression analysis (16). Logistic regression analysis extends the techniques of multiple regression analysis to research situations in which the outcome variable is categorial (17). The least absolute shrinkage and selection operator (LASSO) regression is a shrinkage and variable selection method for regression models, which has been used to determine the variables of ischemic stroke, Alzheimer's disease, COVID-19, and lymph node metastasis (18–22). Nomogram can help present the risk degree of evidence-based outcomes, and the corresponding mathematical equation addresses the impact of risk factors associated with diseases (23, 24). Many studies have identified the risk factors by LASSO regression or multivariate logistic regression and attempted to visualize the incidence and probability of BPD (25, 26). In this study, we sought out to screen risk prediction factors based on these two methods multivariate logistic regression analysis and LASSO logistic regression analysis. The probability of BPD in a premature infant was visualized and compared by evaluating the discrimination performance.

## Materials and methods

### Eligibility criteria

Premature infants with BPD less than 32 weeks of gestational age had been admitted to the Neonatal Intensive Care Unit of Linyi Central Hospital. This study enrolled preterm infants with gestational age less than 32 weeks. At the same time, we collected demographic, clinical and laboratory data. This study enrolled 237 cases of premature infants. This study protocol was reviewed and approved by the Research Ethics Commission of Linyi Central Hospital (No. LCH-LW-202208).

In this study, 36 cases were excluded because of death within 28 days after birth (*n* = 16), maternal mental retardation (*n* = 1), maternal psychiatric abnormality (*n* = 1), aggravation (*n* = 6), and transfer (*n* = 12). The number of premature infants who met the inclusion criteria was 237, and the cases were categorized into training set (*n* = 189) and validation set (*n* = 48). The training set was used to select potential risk factors by univariate logistic regression analysis followed by multivariable logistic regression analysis and LASSO regression analysis. Nomogram model was generated and validated using data from validation cohort. There were no significant differences in demographic, clinical and laboratory results between training dataset and validation dataset (Table 1).

**Table 1:** Demographic, clinical, and laboratory information of BPD and non-BPD patients.

Variables	Overall set	Training set	Validation set	*t/χ^2^/Z*	*P*-values
	(*n* = 237)	(*n* = 189)	(*n* = 48)		
**Puerperal women**					
Age, year, mean ± SD	31.88 ± 5.20	32.06 ± 5.36	31.15 ± 4.50	1.092	0.276
Height, cm, mean ± SD	160.98 ± 4.59	160.98 ± 4.42	160.98 ± 5.24	0.007	0.995
Weight, kg, mean ± SD	73.65 ± 12.37	73.02 ± 11.75	76.11 ± 14.45	−1.546	0.123
BMI, kg/m^2^, mean ± SD	28.42 ± 4.59	28.17 ± 4.29	29.40 ± 5.56	−1.668	0.097
PIH, *n* (%)	56 (23.6)	46 (24.3)	10 (20.8)	0.261	0.610
Diabetes, *n* (%)	38 (16.0)	31 (16.4)	7 (14.6)	0.094	0.759
Low amniotic fluid, *n* (%)	29 (12.2)	22 (11.6)	7 (14.6)	0.309	0.578
PROM, *n* (%)	82 (34.6)	59 (31.2)	23 (47.9)	4.718	0.030*
Birth order, M [25%Q,75%Q]	3.0 [2.0,4.0]	3.0 [2.0,4.0]	2.5 [2.0,4.0]	−1.011	0.312
Multiple births, *n* (%)	65 (27.4)	50 (26.5)	15 (31.3)	0.442	0.506
ACT, *n* (%)	173 (73.0)	138 (73.0)	35 (72.9)	0.000	0.989
Caesarean, *n* (%)	212 (89.5)	167 (88.4)	45 (93.8)	1.179	0.278
**New infants**					
Gender (Male), *n* (%)	118 (49.8)	91 (48.1)	27 (56.3)	1.005	0.316
Gestational age, M [25%Q,75%Q]	30.42 [29.29,31.57]	30.43 [29.14,31.57]	30.43 [29.40,31.29]	−0.385	0.700
Birth weight, kg, mean ± SD	1.38 ± 0.31	1.38 ± 0.30	1.40 ± 0.33	−0.321	0.748
SGA, *n* (%)	23 (9.7)	16 (8.5)	7 (14.6)		0.271
1 min Apgar Scores (<8), M [25%Q,75%Q]	8.0 [7.0,8.0]	8.0 [7.0,8.0]	8.0 [6.3,8.0]	−0.280	0.779
5 min Apgar Scores (<8), M [25%Q,75%Q]	9.0 [8.0,9.0]	9.0 [8.0,9.0]	9.0 [8.0,9.0]	−0.529	0.596
Postnatal asphyxia, *n* (%)	109 (46.0)	88 (46.6)	21 (43.8)	0.122	0.727
IV, *n* (%)					
No IV, *n* (%)	17 (7.2)	15 (7.9)	2 (4.2)	−1.013	0.311
≤ 7, *n* (%)	105 (44.3)	78 (41.3)	27 (56.3)		
> 7, *n* (%)	115 (48.5)	96 (50.8)	105 (44.3)		
NRDS grades					
No NRDS, *n* (%)	59 (24.9)	48 (25.4)	11 (22.9)	−0.788	0.431
Grade I–II, *n* (%)	107 (45.1)	80 (42.3)	27 (56.3)		
Grade III–IV, *n* (%)	71 (30.0)	61 (32.3)	10 (20.8)		
PS application					
No application, *n* (%)	123 (51.9)	95 (50.3)	28 (58.3)	−1.112	0.266
PS, *n* (%)	62 (26.2)	50 (26.5)	12 (25.0)		
PS + Budesonide	52 (21.9)	44 (23.3)	8 (16.7)		
Blood transfusion ≥2 times, *n* (%)	78 (32.9)	59 (31.2)	19 (39.6)	1.213	0.271
HGB, g/L, mean ± SD	200.59 ± 29.43	200.14 ± 29.10	202.35 ± 30.98	−0.464	0.643
WBC, 10^9^/L, M [25%Q, 75%Q]	12.04 [9.07,17.13]	12.04 [8.85,16.89]	12.14 [9.61,18.00]	−0.571	0.568
PLT count, 10^9^/L, mean ± SD	243.72 ± 72.77	244.33 ± 74.14	241.33 ± 67.82	−0.038	0.970
PCT, μg/L, M [25%Q, 75%Q]	0.97 [0.21,6.24]	1.04 [0.21,6.42]	0.65 [0.23,5.06]	−0.111	0.912
PT, s, M [25%Q, 75%Q]	14.40 [13.30,15.60]	14.40 [13.30,15.6]	14.65 [13.60,15.60]	−0.691	0.490
APTT, s, M [25%Q, 75%Q]	41.00 [37.10,44.80]	41.00 [36.65,45.00]	40.90 [38.53,44.58]	−0.823	0.411
FBG, μg/L, M [25%Q, 75%Q]	1.47 [1.21,1.77]	1.48 [1.23,1.82]	1.38 [1.08,1.64]	−1.841	0.066
GPT, IU/L, M [25%Q, 75%Q]	6.00 [4.00,9.50]	7.00 [4.00,10.00]	6.00 [4.00,8.75]	−1.217	0.224
DBIL, μmol/L, mean ± SD	7.60 ± 1.96	7.59 ± 1.97	7.61 ± 1.95	−0.052	0.959
IBIL, μmol/L, M [25%Q, 75%Q]	7.83 [6.70,8.83]	52.26 [32.72,68.60]	44.06 [26.71,60.60]	−1.379	0.168
CKMB, U/L, M [25%Q, 75%Q]	41.00 [26.00,80.00]	41.00 [26.00,77.50]	39.50 [27.00,103.3]	−0.279	0.780
ALBI, g/L, mean ± SD	29.97 ± 3.05	29.93 ± 3.13	30.08 ± 2.75	−0.299	0.765
GLBI, g/L, mean ± SD	11.78 ± 2.63	11.79 ± 2.72	11.73 ± 2.27	0.138	0.890
CREA, μmol/L, M [25%Q, 75%Q]	73.00 [58.00,84.00]	74.00 [59.50,84.00]	71.50 [53.25,84.75]	−0.358	0.720
UA, μmol/L, M [25%Q, 75%Q]	5.20 [3.90,7.00]	5.35 [4.19,7.10]	4.75 [3.30,6.98]	−1.477	0.140
CO_2_CP, mmol/L, M [25%Q, 75%Q]	17.20 [14.85,19.35]	17.20 [14.65,19.10]	17.55 [15.53,19.95]	−0.686	0.493
AG, mmol/L, mean ± SD	20.61 ± 4.19	20.65 ± 3.97	20.45 ± 5.02	0.294	0.769

Data are presented as the mean ± SD, cases number (proportion, %), and median (M) and quartiles (Q1, Q3). *P*-values, values from the training set compared with those from the validation set.

BPD, bronchopulmonary dysplasia; BMI, body mass index; PIH, pregnancy-induced hypertension; PROM, premature rupture of membranes; ACT, antenatal corticosteroid therapy; IV, intensive ventilation; NRDS, neonatal respiratory distress syndrome; PS, pulmonary surfactant; HGB, hemoglobin; WBC, white blood cell; PLT, platelet; PCT, plateletcrit; PT, prothrombin time; APTT, activated partial thromboplastin time; FBG, fibrinogen; GPT, glutamic pyruvic transaminase; DBIL, direct bilirubin; IBIL, indirect bilirubin; CKMB, creatine kinase MB; ALBI, albumin; GLBI, globulin; CREA, creatinine; UA, blood urea nitrogen; CO_2_CP, carbon dioxide combining power; AG, anion gap.

*P-values less than 0.05.

### Definition of BPD

BPD was defined as a categorical variable: no BPD and BPD (mild, moderate and severe BPD) according to BPD criteria of the National institute of Child Health and Development (NICHD) (27). In this study, premature infants born with gestational age less than 32 weeks were diagnosed with BPD as defined by oxygen support more than 21% of fraction of inspired oxygen (FiO_2_) at 36-week postmenstrual age for at least 28 days. Mild BPD was defined as not receiving supplemental oxygen; Moderate BPD was receiving oxygen less than 30% of FiO_2_; Severe BPD was receiving oxygen support more than 30% FiO_2_ or needing positive-pressure ventilation or nasal continuous positive airway pressure.

### Data collection, filtering, and imputation

In this study, the research collected the potential risk factors, including demographic, clinical, and laboratory information. We removed the variables with the proportion of missing values greater than or equal to 20% in each cohort. The missing data were interpolated using the random forest technique. As for data preprocessing, values of height and weight of puerperal women, prothrombin time, indirect bilirubin, creatine kinase MB, and creatinine were winsorized. The values of white blood cell, activated partial thromboplastin time, glutamic pyruvic transaminase, creatine kinase MB, and blood urea nitrogen were log-transformed; and values of indirect bilirubin and CO_2_CP were transformed by squared root function. The categorical variables included birth order, gestational age, Apgar scores (1 min and 5 min), duration of ventilation (more than 1 week), neonatal respiratory distress syndrome (NRDS), plateletcrit, and fibrinogen. The data in 2019, 2021, and 2022 were used as the training set, and the data in 2020 was used as the validation set. The sample size in each group meets the ratio requirement of 7:3. Due to the limited sample size, the data set was randomly split, and the data set was divided into training set and validation set by a ratio of 8:2.

### Logistic regression analysis and nomogram model development

The candidate risk factors were initially analyzed by univariate logistic regression analysis. Multivariate logistic regression analysis was performed for risk factor selection based on forward stepwise selection with a significance level alpha of 0.05, and the selected factors were used for model 1 establishment. Least absolute shrinkage and selection operator (LASSO) was carried out for factor selection with a significance level alpha of 0.15, used for model 2 establishment. SPSS Software was used to perform Student's *t* test, *χ*^2^, *Z*-test, univariate and multivariate logistic regression analysis. The R package “glmnet” was called for LASSO regression analysis. The nomogram models were developed using the R-package “rms”.

### Model evaluation

The predictive performance of model 1 and model 2 was evaluated using ROC curve and calibration curve using the data from internal validation cohort and external validation cohort. The calibration of the nomogram was accompanied with the Hosmer–Lemeshow test. Harrell's C-index was used to measure the discrimination performance of the model. Model evaluation was carried out using the R-package.

## Results

**Figure 1:**
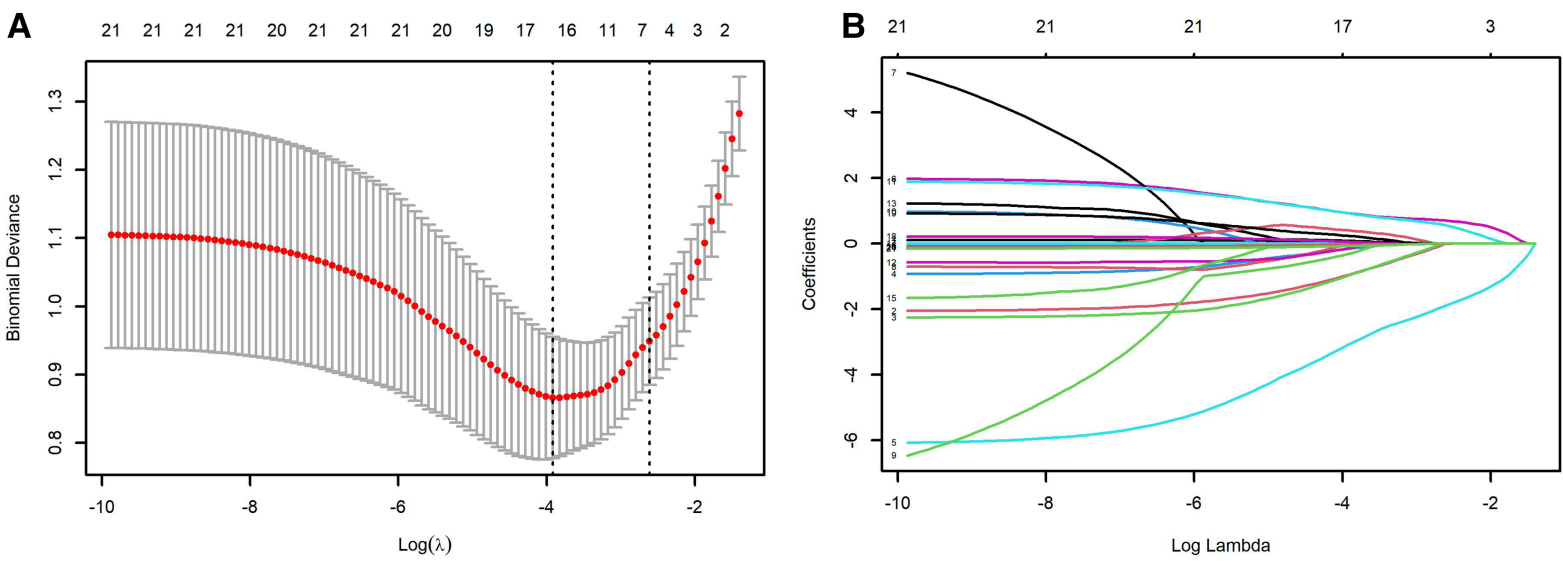
LASSO analysis. (**A**) selection of demographic and clinical characteristics using the LASSO binary logistic regression model; (**B**) optimal parameter (lambda) selection in the LASSO model. The ordinate indicates the target parameter. The upper abscissa represents the number of non-zero coefficients; the lower abscissa represents the log(λ).

Univariable logistic regression analysis revealed maternal age, caesarean, gestational age, birth weight, 1 min Apgar scores (<8), 5 min Apgar scores (<8), postnatal asphyxia, IV, and duration of ventilation (>1 week), NRDS Grade III-IV, pulmonary surfactant (PS) application, PS + budesonide, hemoglobin, prothrombin time, albumin, and GLBI may be associated with BPD. Multivariable logistic regression confirmed that advanced maternal age, gestational age less than 29 weeks, and duration of ventilation more than 1 week may the causative factors or indicators for BPD (Table 2). Caesarean, high birth weight, and increased hemoglobin level may decrease the risk of BPD in preterm infants. The LASSO logistic regression model was analyzed using the R-package glmnet. The optimal *λ* value was determined by cross-validation with the number of folds set to 10. The two dotted lines in Figure 1A represent two values, lambda.min and lambda.1se. Lambda.min is defined as the lambda value of the mean value of the smallest target parameter among the lambda values. As for Lambda.1se, it is the lambda value of the most compact model obtained within a variance range of lambda.min. The red dots represent the mean value of the target parameter, and CI was obtained for the target parameter. The curves in Figure 1B are the trajectory of each independent variable coefficient. As the value of lambda increases, the number of independent variables entering the model decreases. When lambda.1se was selected, the variables delivery, weight and age of premature infants, intensive ventilation more than 7 days, hemoglobin, and albumin were included in the establishment of a risk prediction model 2.

**Table 2:** Univariate and multivariable logistic regression analysis using the training dataset for the development of BPD.

Variables	Training set (*n* = 189)		Univariate logistic regression analysis				Multivariable logistic regression analysis			
	Non-BPD (*n* = 126)	BPD (*n* = 63)	OR	95% CI	Wald *χ^2^*	*P*-value	OR	95% CI	Wald *χ^2^*	*P*-value
**Puerperal women**										
Age, year, mean ± SD	31.37 ± 5.67	33.46 ± 4.65	1.08	1.02, 1.15	6.239	0.012	1.13	1.04, 1.24	7.339	0.007
Height, cm, mean ± SD	160.79 ± 4.68	161.33 ± 4.27	1.03	0.96, 1.10	0.634	0.426				
Weight, kg, mean ± SD	73.39 ± 11.29	72.42 ± 12.39	0.99	0.97, 1.02	0.293	0.588				
BMI, kg/m^2^, mean ± SD	28.39 ± 4.19	27.78 ± 4.40	0.97	0.90, 1.04	0.867	0.966				
PIH, *n* (%)	32 (25.4)	14 (22.2)	0.84	0.41, 1.72	0.230	0.632				
Diabetes, *n* (%)	14 (19.0)	7 (11.1)	0.53	0.22, 1.31	1.886	0.170				
Low amniotic fluid, *n* (%)	14 (11.1)	8 (12.7)	1.16	0.46, 2.94	0.103	0.749				
PROM, *n* (%)	41 (32.5)	18 (28.6)	0.83	0.43, 1.61	0.308	0.579				
Birth order (≥3)	72 (57.1)	37 (58.7)	1.07	0.58, 1.97	0.043	0.835				
Multiple births, *n* (%)	38 (30.2)	12 (19.0)	0.55	0.26, 1.14	2.622	0.105				
ACT, *n* (%)	95 (75.4)	43 (68.3)	0.70	0.36, 1.37	1.083	0.298				
Caesarean, *n* (%)	117 (92.9)	50 (79.4)	0.30	0.12, 0.74	6.848	0.009	0.14	0.03, 0.61	6.996	0.008
**New infants**										
Gender (Female), *n* (%)	71 (56.3)	27 (42.9)	0.58	0.32, 1.07	3.037	0.081				
Gestational age <29 weeks, *n* (%)	9 (7.1)	31 (49.2)	12.59	5.44, 29.14	35.034	<0.001	3.40	1.17, 9.85	5.079	0.024
Birth weight, kg, mean ± SD	1.49 ± 0.27	1.16 ± 0.23	0.006	0.001, 0.029	38.602	<0.001	0.04	0.01, 0.29	10.066	0.002
SGA, *n* (%)	9 (7.1)	7 (11.1)	1.63	0.58, 4.59	0.841	0.359				
1 min Apgar Scores (<8), *n* (%)	51 (58.6)	36 (41.4)	1.96	1.06, 3.62	4.638	0.031				
5 min Apgar Scores (<8), *n* (%)	11 (44.0)	14 (56.0)	2.99	1.27, 7.04	6.255	0.012				
Postnatal asphyxia, *n* (%)	52 (41.3)	36 (57.1)	1.90	1.03, 3.50	4.205	0.040				
IV (>7 days), *n* (%)	6 (4.8)	12 (19.0)	4.71	1.68, 13.23	8.631	0.003				
Duration of ventilation >1 week, *n* (%)	44 (34.9)	52 (82.5)	8.81	4.18, 18.59	32.637	<0.001	3.70	1.31, 10.43	6.120	0.013
NRDS grades										
No NRDS, *n* (%)	38 (30.2)	10 (15.9)	–	–	–	–				
Grade I–II, *n* (%)	55 (43.7)	25 (39.7)	1.73	0.74, 4.01	1.619	0.203				
Grade III–IV, *n* (%)	33 (26.2)	28 (44.4)	3.22	1.37, 7.62	7.126	0.008				
PS application										
No application, *n* (%)	77 (61.1)	18 (28.6)	–	–	–	–				
PS, *n* (%)	25 (19.8)	25 (39.7)	4.28	2.01, 9.11	14.22	<0.001				
PS + Budesonide	24 (19.0)	20 (31.7)	3.56	1.63, 7.81	10.085	0.001				
Blood transfusion ≥2 times, *n* (%)	35 (27.8)	24 (38.1)	1.60	0.84, 3.04	2.067	0.151				
HGB, g/L, mean ± SD	206.71 ± 28.93	187.00 ± 24.82	0.98	0.96, 0.99	17.664	<0.001	0.97	0.95, 0.98	14.504	<0.001
WBC, lnc, mean ± SD	2.50 ± 0.46	2.54 ± 0.61	1.16	0.64, 2.10	0.249	0.618				
PLT count (10^9^/L), mean ± SD	250.43 ± 72.42	232.13 ± 76.60	1.00	0.99, 1.00	2.538	0.111				
PCT					3.096	0.377				
≤0.5 μg/L, n (%)	64 (71.9)	25 (28.1)	–	–	–	–				
0.5–2 μg/L, n (%)	13 (56.5)	10 (43.5)	1.97	0.77, 5.07	1.975	0.160				
2–10 μg/L, n (%)	27 (60.0)	18 (40.0)	1.71	0.80, 3.63	1.928	0.165				
>10 μg/L, n (%)	22 (66.7)	10 (33.3)	1.16	0.48, 2.80	0.114	0.735				
PT, s, mean ± SD	14.51 ± 1.96	15.25 ± 2.95	1.14	1.00, 1.30	3.870	0.049				
APTT, ln, mean ± SD	3.71 ± 1.67	3.73 ± 0.14	2.22	0.33, 14.82	0.680	0.410				
FBG (Q1: ≤1.2 μg/L), n (%)	41 (27.7)	22 (53.7)	3.02	1.48, 6.16	9.28	0.002				
GPT, ln, mean ± SD	1.89 ± 0.65	1.81 ± 0.69	0.84	0.54, 1.33	0.536	0.464				
DBIL, μmol/L, mean ± SD	7.54 ± 2.14	7.70 ± 1.59	1.04	0.89, 1.22	0.254	0.615				
IBIL, Sqart, mean ± SD	7.03 ± 1.70	6.95 ± 1.52	0.97	0.81, 1.17	0.098	0.755				
CKMB, ln, mean ± SD	3.87 ± 0.83	3.91 ± 0.86	1.06	0.74, 1.52	0.090	0.764				
ALBI, g/L, mean ± SD	30.66 ± 2.98	28.49 ± 2.94	0.77	0.68, 0.87	17.93	<0.001				
GLBI, g/L, mean ± SD	12.22 ± 2.74	10.93 ± 2.49	0.82	0.71, 0.93	9.159	0.020				
CREA, μmol/L, mean ± SD	72.88 ± 17.65	71.39 ± 19.36	1.00	0.98, 1.01	0.285	0.594				
UA, ln, mean ± SD	1.66 ± 0.43	1.66 ± 0.42	1.02	0.50, 2.08	0.003	0.958				
CO_2_CP, Sqrt., mean ± SD	4.13 ± 0.39	4.16 ± 0.37	1.30	0.59, 2.85	0.419	0.518				
AG, mmol/L, mean ± SD	20.84 ± 4.10	20.28 ± 3.71	0.97	0.89, 1.04	0.838	0.360				

Data are presented as the mean ± SD, or cases number (proportion, %) after the indexes were preprocessed. *P*-values, values from the BPD set compared with those from non-BPD set.

BPD, bronchopulmonary dysplasia; BMI, body mass index; PIH, pregnancy-induced hypertension; PROM, premature rupture of membranes; ACT, antenatal corticosteroid therapy; IV, intensive ventilation; NRDS, neonatal respiratory distress syndrome; PS, pulmonary surfactant; HGB, hemoglobin; WBC, white blood cell; PLT, platelet; PCT, plateletcrit; PT, prothrombin time; APTT, activated partial thromboplastin time; FBG, fibrinogen; GPT, glutamic pyruvic transaminase; DBIL, direct bilirubin; IBIL, indirect bilirubin; CKMB, creatine kinase MB; ALBI, albumin; GLBI, globulin; CREA, creatinine; UA, blood urea nitrogen; CO_2_CP, carbon dioxide combining power; AG, anion gap.

**Figure 2:**
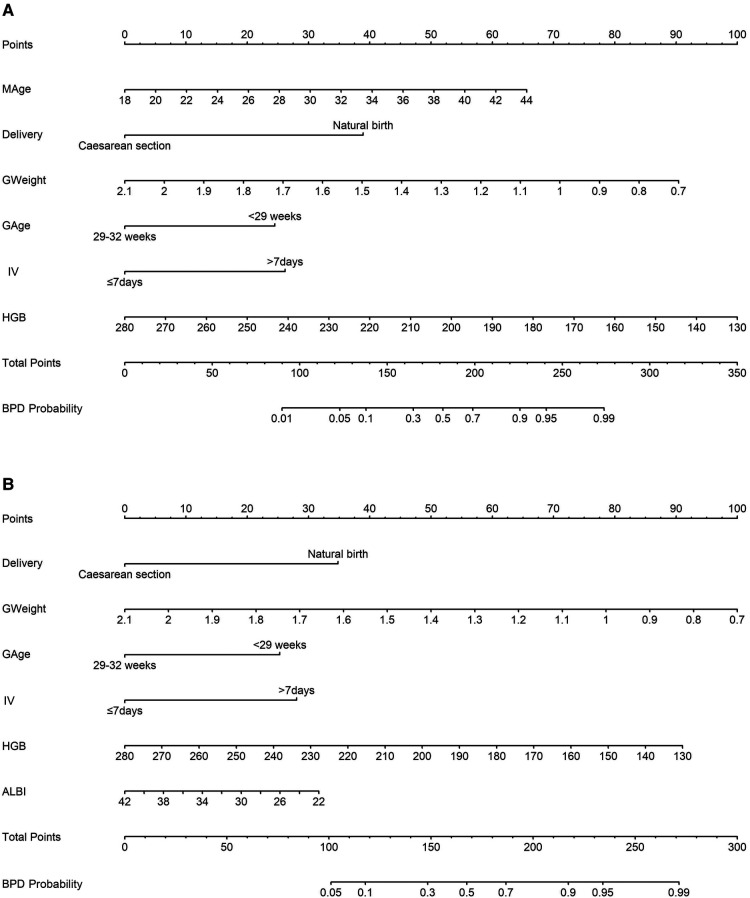
Nomogram models estimating the probability of bronchopulmonary dysplasia (BPD) in a premature infant. (**A**) model 1 incorporates maternal age (MAge), delivery mode, the birth weight and age of premature infants, application of intensive ventilation (IV), and hemoglobin (HGB) level; (**B**) model 2 includes delivery mode, the birth weight and age of premature infants, application of intensive ventilation (IV), hemoglobin level (HGB), and albumin level (ALBI).

**Figure 3:**
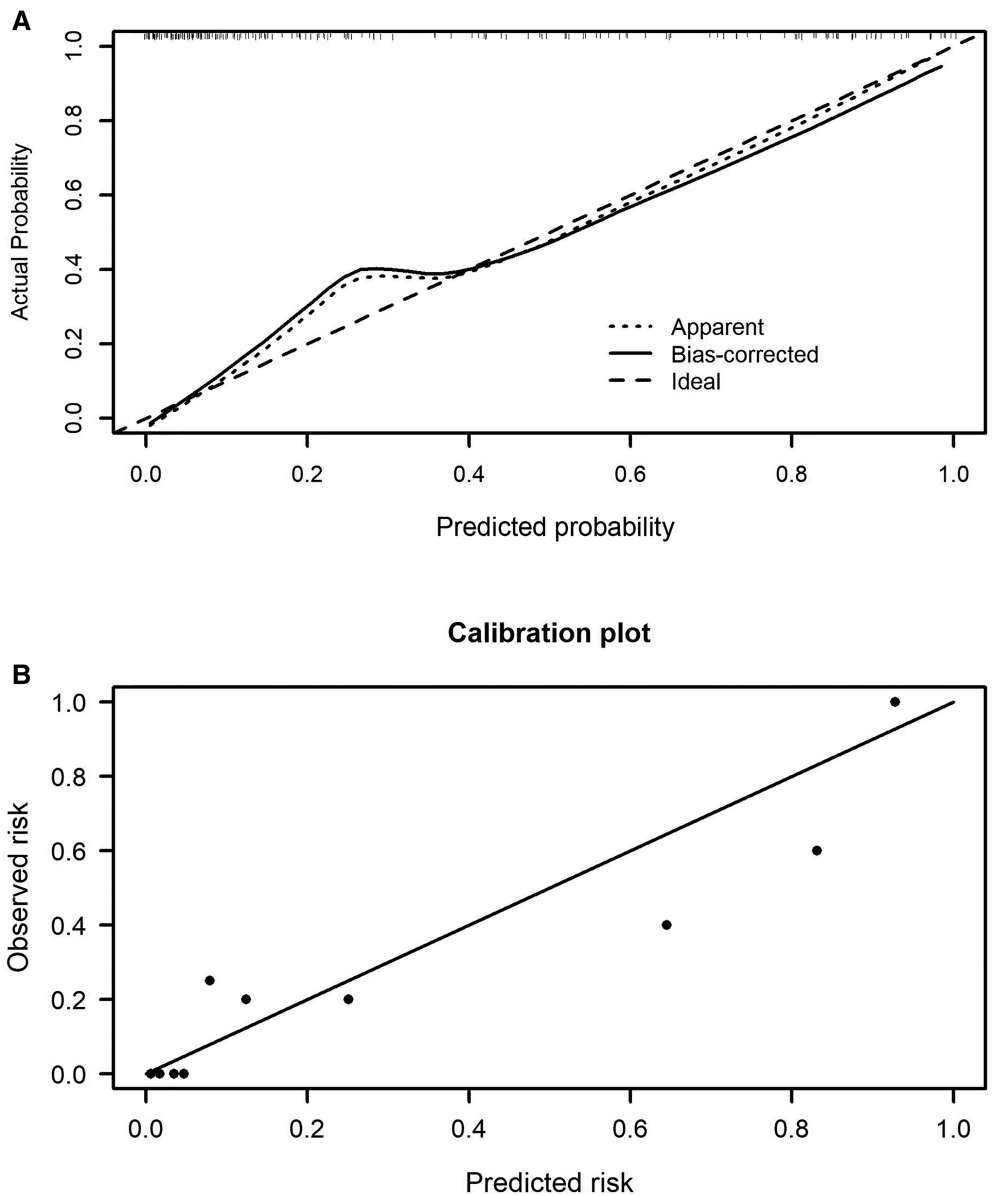
Calibration curves of the nomogram model 1 in the training cohort (**A**) and validation cohort (**B**).

**Figure 4:**
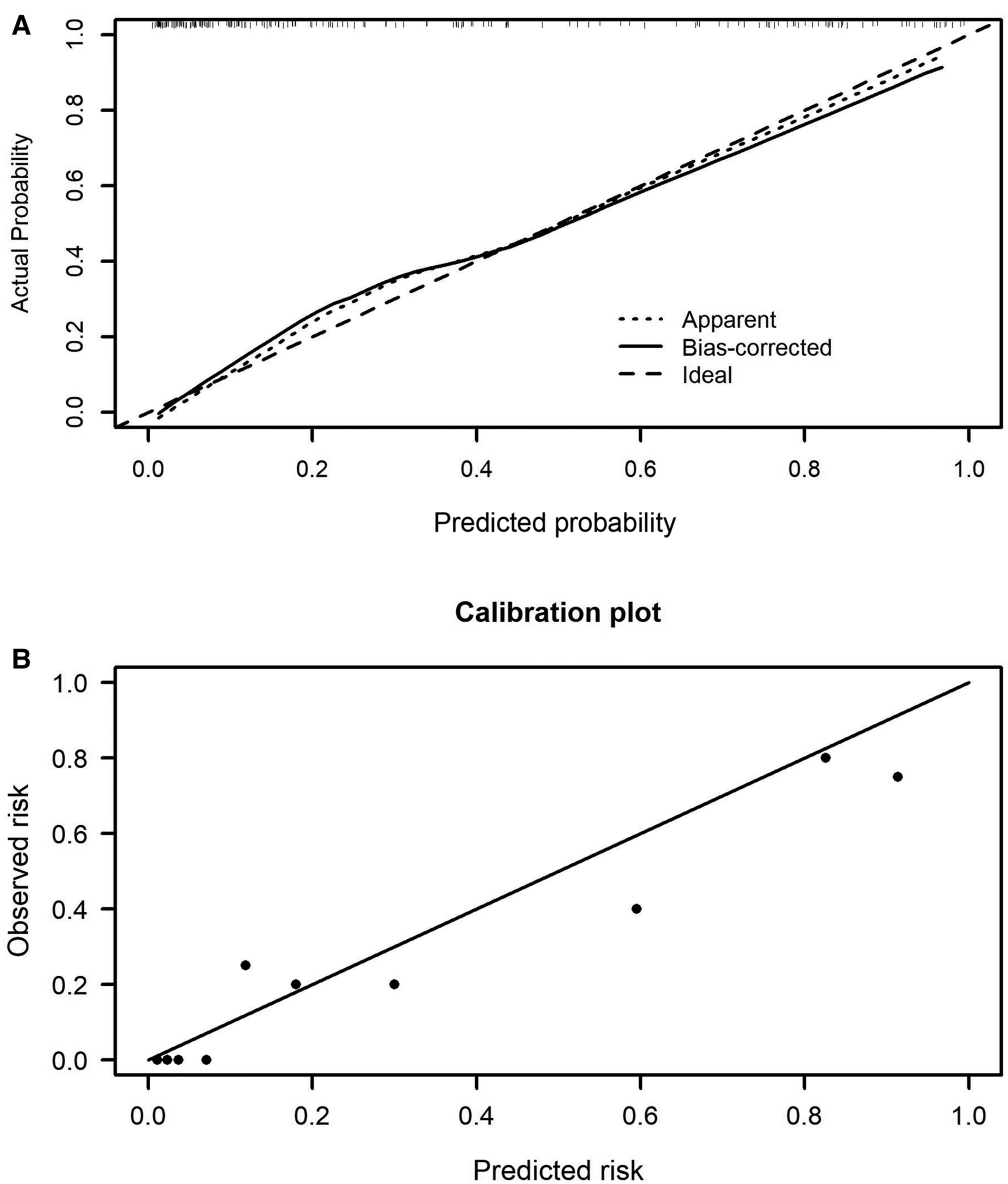
Calibration curves of the nomogram model 2 in the training cohort (**A**) and validation cohort (**B**).

The prediction model 1 and model 2 were presented in the nomogram (Figures 2A,B). For model 1, formula for calculating the probability of BPD was computed as: Logit(P|BPD) = ln(P/1 − P) = 5.690 + 0.126*MAge − 1.943*Delivery − 3.226*GWeight + 1.223*GAge + 1.308*intensive ventilation − 0.033*hemoglobin; As for model 2, Logit(P|BPD) = ln(P/1 − P) = 11.2260 − 1.5403* Delivery − 3.1594*GWeight + 1.1190*GAge + 1.2411*intensive ventilation − 0.0269*hemoglobin − 0.0701*albumin, where MAge is maternal age, GWeight and GAge are the birth weight and age of premature infants, respectively. The calibration curve of the nomogram model 1 for the probability of BPD demonstrated good agreement between prediction and observation between prediction and observation in the training cohort and validation cohort (Figures 3A,B). The Hosmer–Lemeshow test (HL) yielded a nonsignificant statistic (*P* = 0.6823) in training cohort and a nonsignificant difference in validation cohort (*P* = 0.6920). The calibration curve of the nomogram model 2 for the probability of BPD demonstrated good agreement between prediction and observation in the training cohort and validation cohort (Figures 4A,B). The Hosmer–Lemeshow test (HL) yielded a nonsignificant statistic (*P* = 0.5331) in training cohort and a nonsignificant difference in validation cohort (*P* = 0.7796).

**Figure 5:**
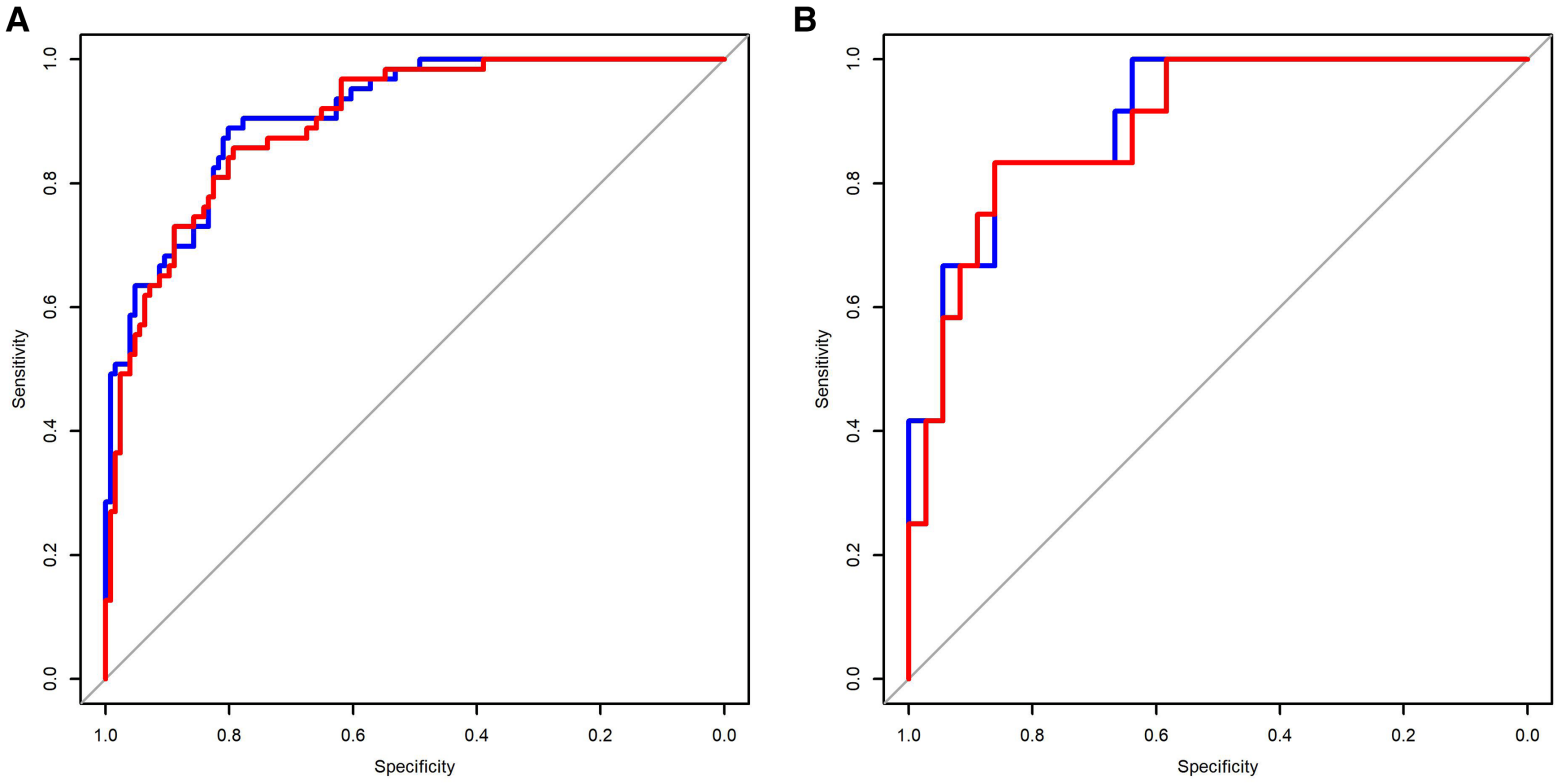
Comparison of the predictive sensitivity and specificity of model 1 and model 2 using the training data (**A**) and validation data (**B**). Model 1 (red line); model 2 (blue line).

**Figure 6:**
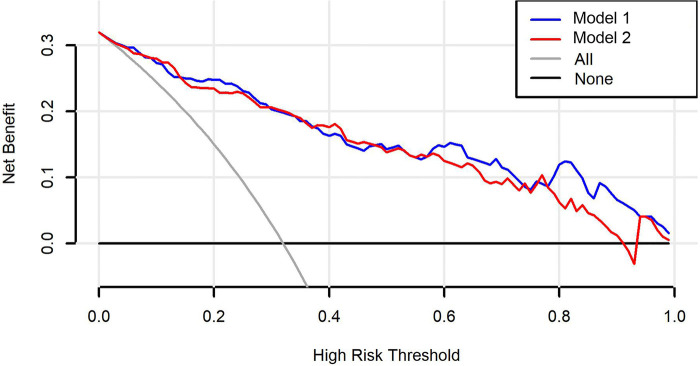
Decision curve analysis (DCA) curves for the model 1 and model 2. The black line is the hypothesis that no patients; the gray line represents the hypothesis that all premature infants have BPD.

The C-indexes (AUC) for the prediction nomogram model 1 and model 2 was 0.910 and 0.899 in the training cohort, respectively (Figures 5A,B). The area under the curve (AUC) value of the nomogram model 1 in the validation cohort was 0.9051, and model 2 was 0.8935. LASSO method has a slightly lower AUC comparing to stepwise method. DCA was then applied to compare the clinical usefulness and benefits between model 1 and model 2. The DCA curves of model 1 and model 2 showed indistinct net benefits across a wide range of threshold probability in the training cohort, indicating both model 1 and model 2 possesses clinical usefulness (Figure 6).

## Discussion

Analysis of 189 neonates revealed that 28-day-old neonates were prospectively predicted at the risk of BPD, which may be associated with advanced maternal age, caesarean, gestational age less than 29 weeks, birth weight, 1 min Apgar scores (less than 8), 5 min Apgar scores (less than 8), postnatal asphyxia, intensive ventilation application, duration of ventilation (more than 1 week), NRDS grade III-IV, PS application, PS and budesonide application, decreased hemoglobin, increased prothrombin time, and decreased albumin and GLBI. Risk factors identified by multivariate and LASSO logistic regression analysis were considered early predictors for the development of risk prediction model for BPD. Stepwise and LASSO analysis all selected delivery, birth weight, birth age, intensive ventilation application, and hemoglobin level as predictors. It is clear that LASSO selection method showed better prediction accuracy and interpretability compared to stepwise method.

The maternal parameters were analyzed for selecting risk factors of BPD. The main causes of BPD are advanced maternal age and delivery by caesarean section. There is an ongoing trend in China and developed countries that delayed childbearing shows no signs of diminishing although preterm newborns born to women of increasing maternal age are reported with multiple adverse birth outcomes like BPD (28). For very preterm infants, increasing maternal age is not significantly associated with neonatal mortality or major morbidity (29). Instead, younger maternal age may increase the risk of severe intraventricular hemorrhage in very preterm infants (29). Delivery by caesarean section remained significantly associated with the decreased occurrence of BPD. It is conceivable that these preterm infants born by caesarean section were exposed to postnatal antibiotics, preventing multiple respiratory disorders and lung injury (2).

This study analyzed various antenatal, perinatal, and postnatal factors that may contribute to the development of BPD. Preterm infants born at <29 weeks of gestational age had an increased incidence of BPD. Infants weight was significantly lower in BPD than non-BPD group. Although intensive ventilation and prolonged ventilation can cause lung injury and are risk factors for BPD, lung-protective ventilation is still an important strategy for the current clinical resuscitation of critically ill preterm neonates. Incomplete differentiation of alveolar type 2 cells causes a lack of pulmonary surfactant occurring fairly fate in gestation (30). Pulmonary surfactant replacement therapy and budesonide application were believed to increase the incidence of BPD. However, contrary findings were witnessed with the introduction of the routine application of pulmonary surfactant and budesonide (31–34). It was believed that our study is a retrospective and non-randomized controlled study. Premature infants receiving endotracheal application of pulmonary surfactant and budesonide were mainly with lower body weight and younger gestation age compared to other studies, which may cause a higher incidence of BPD.

BPD severity is greatly associated with Apgar scores at 1 min and 5 min (35, 36). Premature infants diagnosed with BPD were registered with lower 5 min Apgar scores compared with non-BPD. Postnatal asphyxia was linked to the development of BPD as evidenced by our analysis and other studies (37–40). In our preterm infants, NRDS, especially NRDS grade III–IV, is an important cause of BPD. Laboratory examination revealed that premature infants with BPD showed increased prothrombin time and decreased levels of hemoglobin, albumin and globulin. Fetal hemoglobin decreases early, indicating the reduction of endogenous blood component, which has been proposed as a predictive value for BPD development (41). The levels of albumin and globulin have been considered biomarkers associated with BPD in other studies (42).

Multivariate and LASSO logistic regression analysis was carried out to select the predictors for BPD. It is worth noting that LASSO differently selected albumin as the predictors instead of maternal age compared to stepwise method. Both stepwise and LASSO-based nomogram models exhibited good discrimination. Model 1 by stepwise method had a favorable discrimination performance with an AUC of 0.9051, compared to 0.8935 for LASSO logistic regression model 2, analyzed with the validation dataset. A retrospective analysis included risk factors birth weight, gestational age, gender, et al. for generating risk scoring system, of which a sensitivity was 65%–90.3% and a specificity was 77.8%–88% (36, 43). The strengths of our models included a more favorable discrimination performance, comparison of two predictor selection methods stepwise and LASSO logistic regression analysis, and external validation of the risk factors. The established prediction models could be used to predict the probability of BPD for premature infants by using the scoring formulae that were proposed based on maternal age, delivery mode, the birth weight and age of premature infants, application of intensive ventilation, hemoglobin level, and albumin level. The established models may help the clinicians to early diagnose the disease, design therapy project and estimate prognosis. However, our study was limited in selection bias and a relatively small sample of BPD from a single medical center.

## Conclusions

This study of risk scoring for BPD supports that the probability of BPD could be predicted for premature infants by maternal age, delivery options, birth weight, birth age, invasive ventilation, hemoglobin and albumin identified by stepwise and LASSO logistic regression analysis. The developed nomogram model 1 and model 2 provided risk predictors for BPD, and explain the potential detriments for premature infants. However, further larger samples from multiple medical centers are required for external validation.

## Data Availability

The original contributions presented in the study are included in the article/Supplementary Material, further inquiries can be directed to the corresponding author.
